# Association Study of *BDNF*, *SLC6A4*, and *FTO* Genetic Variants with Schizophrenia Spectrum Disorders

**DOI:** 10.3390/jpm13040658

**Published:** 2023-04-12

**Authors:** Aneta Bednarova, Viera Habalova, Michaela Krivosova, Matteo Marcatili, Ivan Tkac

**Affiliations:** 1 2nd Department of Psychiatry, Faculty of Medicine, Pavol Jozef Safarik University, Louis Pasteur University Hospital, 041 90 Kosice, Slovakia; 2 Department of Medical Biology, Faculty of Medicine, Pavol Jozef Safarik University, 040 11 Kosice, Slovakia; viera.habalova@upjs.sk; 3 Biomedical Centre Martin, Jessenius Faculty of Medicine in Martin, Comenius University in Bratislava, 036 01 Martin, Slovakia; michaela.krivosova@uniba.sk; 4 Department of Mental Health and Addiction, Fondazione IRCCS San Gerardo dei Tintori, 209 00 Monza, Italy; matteo.marcatili@irccs-sangerardo.it; 5 4th Department of Internal Medicine, Faculty of Medicine, Pavol Jozef Safarik University, Louis Pasteur University Hospital, 041 90 Kosice, Slovakia; ivan.tkac@upjs.sk

**Keywords:** schizophrenia, polymorphism, high resolution melting, 5-HTTLPR, rs9939609, rs6265, rs962369

## Abstract

Schizophrenia spectrum disorders (patients with a diagnosis of schizophrenia, schizotypal, and delusional disorders: F20-F29 according to International Classification of Diseases 10th revision (ICD-10)) are considered highly heritable heterogeneous psychiatric conditions. Their pathophysiology is multifactorial with involved dysregulated serotonergic neurotransmission and synaptic plasticity. The present study aimed to evaluate the association of *SLC6A4* (5-HTTLPR), *FTO* (rs9939609), and *BDNF* (rs6265, rs962369) polymorphisms with schizophrenia spectrum disorders in Slovak patients. We analyzed the genotypes of 150 patients with schizophrenia, schizotypal, and delusional disorders and compared them with genotypes from 178 healthy volunteers. We have found a marginally protective effect of LS + SS genotypes of 5-HTTLPR variant of the serotonin transporter *SLC6A4* gene against the development of schizophrenia spectrum disorders, but the result failed to remain significant after Bonferroni correction. Similarly, we have not proven any significant association between other selected genetic variants and schizophrenia and related disorders. Studies including a higher number of subjects are warranted to reliably confirm the presence or absence of the studied associations.

## 1. Introduction

Globally, it is estimated that more than 24 million people suffer from schizophrenia spectrum disorders [1], and the lifetime prevalence of this broadly defined psychotic disorders group has been reported with a range between 0.5% and 2.3% [2,3,4,5,6,7]. The most studied psychotic disorder is undoubtedly schizophrenia. Its onset typically occurs in late adolescence or early adulthood, and growing evidence from clinical and epidemiological studies suggests that schizophrenia may reflect a disturbance of neurodevelopment. Although the prevalence is lower compared to other psychiatric conditions such as affective disorders and anxiety [8,9], it is associated with serious physical illnesses, fundamentally lowers the quality of life, and elevates the risk of suicide [1,10]. Schizophrenia is a complex, heterogeneous behavioral and cognitive syndrome with a high heritability reaching 60–80% [10]. It is a highly polygenic disorder; most of the inter-individual variation associated with schizophrenia risk is genetic, involving large numbers of common alleles, rare copy number variants (CNVs), and rare coding variants [11,12,13,14].

Brain-derived neurotrophic factor (BDNF) is the most common nerve growth factor secreted in the brain. It is a key regulator of synaptic transmission and plasticity, hence vital for various cognitive functions and implicated in schizophrenia spectrum disorders [15]. Several meta-analyses have previously found a higher risk for schizophrenia in Met/Met carriers of non-synonymous Val66Met polymorphism in *BDNF* [16,17], although others have not reported any association [18,19,20,21].

The 5-HTTLPR variant in the promotor area of the serotonin transporter gene *SLC6A4* has long been considered a candidate variant for the pathogenesis of schizophrenia for different reasons. It has been reported that levels of serotonin, mRNA of *SLC6A4*, and serotonin transporter protein were significantly different in schizophrenic patients compared with healthy controls [22,23,24,25]. Furthermore, many second and third generation antipsychotic compounds act at multiple sites modulating serotoninergic transmission, thus suggesting that serotonin might be involved in the pathogenesis of schizophrenia [26]. The data on possible associations between the *SLC6A4* 5-HTTLPR variant and schizophrenia are, however, controversial [27,28].

The pathophysiology of psychiatric diseases including schizophrenia is multifactorial, and some evidence also suggests the contribution of chronic inflammatory state and oxidative stress pathways [29]. These mechanisms are essential factors also in other conditions such as atherogenicity, insulin resistance, obesity, and type 2 diabetes, overall, in metabolic syndrome [30]. The fat mass and obesity-associated protein (FTO), encoded by the *FTO* gene, is implicated in adipocyte dysfunction and obesity development. It has been also shown that obesity and cardiovascular diseases contribute to neuropsychiatric diseases development and vice versa [31,32], and the bidirectional relationship can be observed already in adolescence [33,34,35]. *FTO* polymorphisms have been studied for the association with mental diseases, and a recent meta-analysis observed a significant association of rs9939609 with the major depressive disorder [36], but the association with schizophrenia spectrum disorders has not been studied so far.

The present study aimed to evaluate the possible association of selected polymorphisms with the risk for schizophrenia spectrum disorders in a Central European Caucasian population. The polymorphisms in the present study were selected based on the above-mentioned mechanistic connections and previous controversial evidence such as 5-HTTLPR polymorphism in the promotor region of *SLC6A4* and *BDNF* (rs6265; C > T; Val66Met) or based on the lack of association data with schizophrenia spectrum disorders such as *BDNF* (rs962369; T > C) and *FTO* (rs9939609; T > A).

## 2. Materials and Methods

### 2.1. Study Sample

Overall, 150 unrelated patients with a diagnosis of schizophrenia, schizotypal, and delusional disorders (F20-F29 according to International Classification of Diseases 10th revision (ICD-10)) were enrolled in the study. The control group (*n* = 178) was age-, sex-, and ethnicity-matched to the patient’s group. The control group consisted of adult volunteers with no relation to the cases, no known psychiatric disorder, and no history of mental disorders. The participants included in both groups were of Slovak origin (Caucasians). Blood samples were collected from November 2018 to January 2023, and the participation of subjects in testing was voluntary and could be canceled by any individual at any time during the study. This study was approved by the Ethics Committee of the Louis Pasteur University Hospital in Kosice, and all subjects provided written informed consent. The study was conducted in accordance with the principles of the Declaration of Helsinki.

### 2.2. Genotyping

Genotyping of the *FTO* rs9939609 and *BDNF* (rs6265, rs962369) polymorphisms was performed by high-resolution melting analysis in the presence of an unlabeled probe [37]. The *SLC6A4* (5-HTTLPR) gene variants were analyzed by gel electrophoresis after a polymerase chain reaction [38].

### 2.3. Statistical Analysis

Online software SNPstats was used to measure the strength of the relative associations via odds ratios (ORs) with their corresponding 95% confidence intervals (CIs) and a *p*-value [39]. The Hardy–Weinberg equilibrium (HWE) and haplotype association analyses were done by using the same software. For the primary analysis of the possible associations between genetic variants and phenotypes, we used a codominant genetic model. Since four gene variants were evaluated, a Bonferroni corrected *p*-value of 0.05/4 = 0.0125 was considered statistically significant. A *p*-value of <0.05 was considered nominally significant.

## 3. Results

One-hundred and fifty patients with a diagnosis of schizophrenia, schizotypal, and delusional disorders (F20-F29 according to ICD-10) and age-, sex-, and ethnicity-matched 178 control subjects were enrolled in the present study. The demographic characteristics can be found in Table 1 and a list of diagnoses in Table 2. The most frequent diagnoses in our patient’s group were schizophrenia (48.7%), schizoaffective disorders (30%), and acute and transient psychotic disorders (14.7%).

The allelic frequencies and HWE of selected polymorphisms in healthy controls and patients with schizophrenia spectrum disorders are shown in Table 3. The genotype distribution was consistent with HWE among the control subjects for all selected polymorphisms. Haplotype analysis of *BDNF* polymorphisms (rs6265, rs962369) has not confirmed any significant association with schizophrenia spectrum disorders (*p* > 0.05, data are not shown). The most common haplotype was CT with a cumulative frequency 0.5885, and the least common haplotype was TC with cumulative frequency 0.0062.

Table 4 shows the analysis of the association between gene variants and schizophrenia, schizotypal, and delusional disorders under the codominant genetic model. We have found no significant differences in genotype distributions between the patient and control group for *FTO* and *BDNF* genes, but the trend of lower frequency of LS and SS genotypes in *SCL6A4* in the patient’s group compared to controls was observed. Since ORs for LS and SS genotypes were similar (0.59 and 0.69, respectively), we decided to further analyze the association under the dominant model (Table 5). Under the dominant genetic model, the protective effect for carriers of at least one S allele (genotypes LS + SS) was seen (OR = 0.62, 95% CI = 0.39–0.98, *p* = 0.04). A similar nominal statistical significance was reached in the females’ subgroup (LS + SS vs. LL, OR = 0.50, 95% CI = 0.26–0.98, *p* = 0.04) but not in male subjects.

## 4. Discussion

In the present study, we assessed the potential association of four genetic variants *SLC6A4 5-HTTLPR*, *BDNF* rs6265, rs962369, and *FTO* rs9939609, with diagnosis F20-F29 in Slovak patients. We did not confirm any significant association between schizophrenia and related disorders and evaluated polymorphisms under a codominant genetic model.

As serotonin plays an important role in mental diseases including schizophrenia spectrum disorders when dysregulated, there have been many studies looking for an association between genetic polymorphisms of its receptors or a gene *SLC6A4* encoding a serotonin transporter. The most studied variant of *SLC6A4* gene is insertion/deletion polymorphism in a highly polymorphic promoter region 5-HTTLPR. In the present study, we have found slightly less frequent LS + SS genotypes in schizophrenia patients compared to healthy controls, but the *p*-value reached only nominal statistical significance and became not significant after Bonferroni correction for the number of variants examined. The opposite results were recently found in Iran [40]. The results on this topic have been contradictory so far. While some studies did not similarly find any significant association [41,42,43,44,45,46], a French study observed an excess of the transmission of the L allele in a family-based study [47]. Previous studies in Slavic populations also have not proven the association of 5-HTTLPR with schizophrenia [48,49,50]. In a Russian study, there was not found an association with schizophrenia without affective symptoms; however, the frequency of the SS genotype was significantly higher in affective psychosis [48]. Even though some studies have not found a significant association in this unique genetic variant, the interaction with other genes can lead to a significantly higher risk of schizophrenia. In the study of Sáiz et al. [46], the synergistic interaction between 5-HTTLPR and 5-hydroxytryptamine receptor 2A (*HTR2A*) rs6311 polymorphisms in relation to higher susceptibility to schizophrenia was described. Another study found a marginal association for family history of schizophrenia after interaction analysis between 5-HTTLPR and tumor necrosis factor (*TNF*) rs61525 polymorphisms [42]. The effect of 5-HTTLPR polymorphism on the development of schizophrenia was assessed also in meta-analyses [51,52,53]; however, no significant association was found with schizophrenia, except for the Indian subgroup in the latest study.

Regarding the association of *FTO* genetic variants with schizophrenia spectrum disorders, we have not confirmed the association under the codominant genetic model analyzed in our study. In the literature, this gene was mostly associated with metabolic syndrome, antipsychotic-induced weight gain, and body mass index (BMI) increase in patients with schizophrenia [54]. A significant association was found between rs9939609 and the occurrence of metabolic syndrome in schizophrenia [55], with higher BMI in chronically treated patients [56] and with weight gain after 6 months of risperidone administration [57].

We have not proven a statistically significant association of two common *BDNF* gene variants (rs6265, rs962369) with schizophrenia spectrum disorders even after association haplotype analysis. The first gene variant has been frequently assessed in numerous studies; however, no consistent conclusion has been reached. Some studies with participants of Slavic origin have found no association between Val66Met polymorphism and schizophrenia [58,59]. In Armenian [60] and Greek studies [61], the Met allele has been suggested as a risk, which was also confirmed in meta-analyses [16,17]. The Met allele was a risk factor for psychosis in Croatian patients with Alzheimer’s disease [62]. However, many European studies [63,64], Asian studies [65,66], and American studies [67,68] have not confirmed these results. There was a study that found a difference between various schizophrenia spectrum disorders on the base haplotype analysis of five *BDNF* variants. The schizoaffective disorder haplotype frequencies were found to be similar to other affective disorders and dissimilar from schizophrenia and healthy controls. However, the Val66Met (rs6265) examined in isolation was only significant in the comparison of all individuals with schizoaffective or major affective disorder to healthy controls [69]. On the other hand, some studies suggested an association between genetic variants and the age of schizophrenia onset. Met allele or Met/Met genotype have been found a risk factor for earlier onset of schizophrenia [60,70,71,72], and one study observed an earlier onset in Val/Met heterozygotes [73].

There are several reasons why there may be inconsistencies among studies looking for an association between genetic polymorphisms and psychoses including schizophrenia: one of the most important factors that can influence the results of genetic association studies is the size of the sample population. Studies with small sample sizes may have low statistical power and may not be able to detect small genetic effects, which can result in false negative findings. Genetic variation can differ between populations, and therefore, associations between genetic polymorphisms and psychoses may vary across different ethnic groups. Studies that include participants from different ethnic backgrounds may produce different results. Psychoses such as schizophrenia are complex disorders that can have multiple underlying causes. It is possible that genetic associations may be more pronounced in certain subtypes of the disorder or individuals with specific symptom profiles. Failure to account for disease heterogeneity can lead to inconsistent results. Variations in study design, such as differences in the criteria for diagnosis or differences in the way that genetic data are analyzed, can impact the results of genetic association studies. Failure to control for these factors can lead to inconsistent results. The studies that report positive findings are more likely to be published than those that report negative findings. This can create a publication bias that can lead to an overestimation of genetic effects.

Personalized medicine for schizophrenia is an area of active research and development. Currently, the standard of care for treating schizophrenia involves a trial-and-error approach, where different medications are tried until one is found that works for the individual patient. However, this approach can be time-consuming and may result in suboptimal outcomes for some patients.

In the future, personalized medicine for schizophrenia could involve the use of biomarkers to identify specific subtypes of the disorder and to guide treatment decisions. For example, researchers are investigating the use of genetic testing to identify individuals who are more likely to respond to certain medications. Additionally, brain imaging techniques may be used to identify specific neural circuits that are disrupted in individual patients, allowing for targeted interventions such as neuromodulation.

Another area of personalized medicine for schizophrenia is the development of tailored psychosocial interventions. This could involve identifying specific cognitive or behavioral deficits in individual patients and designing interventions to address these deficits. For example, computerized cognitive training programs could be used to improve cognitive function in individuals with schizophrenia.

## 5. Conclusions

In conclusion, the present study did not find a significant association between schizophrenia and related disorders and selected gene variants in *SLC6A4*, *BDNF*, and *FTO*. It might have been caused by the fact that the effects of the investigated variants were so small that they could not have been detected in this kind of genetic association study including a relatively low number of patients. Further research approaches are needed to examine the association of this complex psychiatric disorder with genetic variability. The sensitivity to detect even small effects of gene variants will be increased by using GWAS and deriving polygenic risk scores (PRS). PGS are a type of biomarker that can be used to predict an individual’s genetic risk for developing a particular disorder. PRS are calculated by combining information from multiple genetic variants that have been associated with the disorder of interest. The resulting score can be used to identify individuals who are at higher risk for developing the disorder, even if they do not show any symptoms.

## Figures and Tables

**Table 1:** Demographic data of the study subjects.

Age	Controls			Patients		
	Total	Males	Female	Total	Males	Females
Number (%)	178	97 (54.5)	81 (45.5)	150	82 (54.6)	68 (45.4)
Average ± SD	46.7 ± 15.2	43.9 ± 16.0	51.3 ± 15.0	45.6 ± 13.1	42.2 ± 14.0	49.6 ± 10.6
min–max	21–80	21–73	23–80	20–71	20–71	31–71

**Table 2:** List of diagnoses of patients with schizophrenia spectrum disorders.

Code	Diagnosis	Total		Males		Females	
		*n*	%	*n*	%	*n*	%
F20	Schizophrenia	73	48.7	45	54.9	28	41.2
F21	Schizotypal disorder	1	0.7	0	0.0	1	1.5
F22	Persistent delusional disorders	8	5.3	4	4.9	4	5.9
F23	Acute and transient psychotic disorders	22	14.7	16	19.5	6	8.8
F25	Schizoaffective disorders	45	30.0	17	20.7	28	41.2
F29	Unspecified nonorganic psychosis	1	0.7	0	0.0	1	1.5

According to ICD-10.

**Table 3:** Allelic frequencies and HWE of selected polymorphisms in healthy controls and patients with schizophrenia spectrum disorders.

	Allele	Patients		Controls		Controls HWE *p*-Value
		*n*	Frequency	*n*	Frequency	
*SLC6A4* 5-HTTLPR	L	178	0.59	190	0.53	1.00
	S	122	0.41	166	0.47	
*FTO* rs9939609	T	170	0.57	187	0.53	0.88
	A	130	0.43	169	0.47	
*BDNF* rs6265	C	243	0.81	288	0.81	0.47
	T	57	0.19	68	0.19	
*BDNF* rs962369	T	229	0.76	278	0.78	0.19
	C	71	0.24	78	0.22	

HWE—exact test for Hardy–Weinberg equilibrium.

**Table 4:** Genotype distribution of selected polymorphisms in patients with schizophrenia spectrum disorders and healthy controls under a codominant model.

	Genotype	Controls		Patients		OR (95% CI)	*p*-Value
		*n*	(%)	*n*	(%)		
*SLC6A4* 5-HTTLPR	LL	51	(28.6)	59	(39.3)	1.00	0.11
	LS	88	(49.4)	60	(40.0)	0.59 (0.36–0.97)	
	SS	39	(21.9)	31	(20.7)	0.69 (0.38–1.25)	
*FTO* rs9939609	TT	48	(27.0)	47	(31.3)	1.00	0.56
	TA	91	(51.1)	76	(50.7)	0.85 (0.52–1.41)	
	AA	39	(21.9)	27	(18.0)	0.71 (0.37–1.33)	
*BDNF* rs6265	CC	118	(66.3)	101	(67.3)	1.00	0.89
	CT	52	(29.2)	41	(27.3)	0.92 (0.57–1.50)	
	TT	8	(4.5)	8	(5.3)	1.17 (0.42–3.23)	
*BDNF* rs962369	TT	105	(59.0)	87	(58.0)	1.00	0.5
	TC	68	(38.2)	55	(36.7)	0.98 (0.62–1.54)	
	CC	5	(2.8)	8	(5.3)	1.93 (0.61–6.12)	

**Table 5:** The dominant model for *SLC6A4* (5-HTTLPR) polymorphism in the total group and after gender stratification.

	Genotype	Controls n (%)	Patients n (%)	OR (95% CI)	*p*-Value
Total	LL	51 (28.6)	59 (39.3)	1.00	**0.04**
	LS + SS	127 (71.3)	91 (60.7)	**0.62 (0.39–0.98)**	
Males	LL	25 (25.8)	26 (31.7)	1.00	0.38
	LS + SS	72 (74.2)	56 (68.3)	0.75 (0.39–1.43)	
Females	LL	26 (32.1)	33 (48.5)	1.00	**0.04**
	LS + SS	55 (67.9)	35 (51.5)	**0.50 (0.26–0.98)**	

## Data Availability

The data that support the findings of this study are available upon request.
